# Torsion of the Vermiform Appendix in an 18-Day-Old Neonate: A Case Report from Romania and Review of the Literature

**DOI:** 10.3390/reports9020182

**Published:** 2026-06-10

**Authors:** Paul Tchouala Tchakoute, Alin Iuhas, Vlad-Ionuț Nechita, Andrei Vasile Pașcalău, Ion Cosmin Puia

**Affiliations:** 1 Doctoral School of Biomedical Sciences, University of Oradea, 410073 Oradea, Romania; 2 Department of Pediatric Surgery and Orthopedics, Bihor County Emergency Clinical Hospital, 410469 Oradea, Romania; 3 Department of Medical Disciplines, Faculty of Medicine and Pharmacy, University of Oradea, 410087 Oradea, Romania; 4 Department of Fundamental Sciences, Discipline of Medical Informatics, Research Methodology and Data Analysis, Faculty of Nursing and Health Sciences (FAMSS), “Iuliu Hațieganu” University of Medicine and Pharmacy, 400012 Cluj-Napoca, Romania; 5 Department of Morphological Disciplines, Faculty of Medicine and Pharmacy, University of Oradea, 410087 Oradea, Romania; 6 Department of Pathology, Bihor County Emergency Clinical Hospital, 410469 Oradea, Romania; 7 Department of Surgery, “Octavian Fodor” Regional Institute of Gastroenterology and Hepatology, 400162 Cluj-Napoca, Romania; 8 Department of Surgery, “Iuliu Hațieganu” University of Medicine and Pharmacy, 400162 Cluj-Napoca, Romania

**Keywords:** appendiceal torsion, vermiform appendix, neonatal acute abdomen, newborn, appendectomy, case report, Romania

## Abstract

**Background and Clinical Significance:** Torsion of the vermiform appendix is a rare condition with a clinical presentation closely resembling acute appendicitis, while preoperative investigations are of limited value in distinguishing between the two entities. In most cases, the definitive diagnosis is made incidentally during surgery. **Case Presentation**: The authors present the case of an 18-day-old female neonate who presented with marked abdominal distension, diffuse spontaneous and palpation-induced abdominal pain, guarding, and signs of peritoneal irritation. The clinical manifestations and paraclinical findings mimicked a neonatal intestinal obstruction; however, intraoperative exploration revealed a gangrenous vermiform appendix twisted 240° anticlockwise, associated with a fibrinous pseudomembrane and multiple enlarged mesenteric lymph nodes. Although the initial therapeutic strategy was to perform a laparoscopy, severe abdominal distension caused by marked aerocolia necessitated conversion to a supra- and infraumbilical midline laparotomy. We thus describe, to the best of our knowledge, one of the youngest neonatal cases of appendiceal torsion reported in the literature. **Conclusions:** Although rare, appendiceal torsion should be considered in the differential diagnosis of neonatal acute abdomen, and timely surgical exploration is key to achieving a favorable outcome.

## 1. Introduction and Clinical Significance

Appendiceal torsion, first described by Payne in 1918 [1], is a condition characterized by rotation of the vermiform appendix around its longitudinal axis, a phenomenon that results in luminal obstruction and progressive compromise of venous and lymphatic circulation, subsequently followed by impairment of arterial flow [2,3]. The consequence of these hemodynamic alterations is the development of tissue ischemia, with potential progression to necrosis and luminal distension. The clinical presentation frequently mimics acute appendicitis [2,4].

The etiology of appendiceal torsion remains unclear; however, appendiceal torsion can be classified into primary (idiopathic) form, associated with anatomical peculiarities such as appendiceal elongation, a narrow-based mesoappendix, or absence of fixation folds, and secondary form, determined by the presence of local pathological processes [2,3]. Among the causes of secondary forms are obstruction by fecaliths, mucoceles, tumors, foreign bodies, or other lesions that modify the mobility or structure of the appendix [2,5,6,7].

From an epidemiological perspective, appendiceal torsion appears to predominantly affect the pediatric population and adult males, with the male-to-female ratio estimated at approximately 4.5:1 [3,8]. Age distribution remains heterogeneous, with reported cases ranging from a 50-day-old infant to a 79-year-old adult [9,10].

Based on the available literature, appendiceal torsion appears to be a rare condition, with only 82 cases reported worldwide, of which only 34 are pediatric patients. The present case represents, to the best of our knowledge, the first reported pediatric case from Romania and one of the youngest neonatal patients described in the literature to date.

## 2. Case Presentation

An 18-day-old full-term female neonate, the second child of the family, born via cesarean section following an uncomplicated pregnancy with a birth weight of 4200 g, presented to the emergency department with abdominal distension and bilious vomiting of approximately 14 hours’ duration.

On physical examination, the patient demonstrated psychomotor agitation with deterioration of general condition, was afebrile (36.9 °C), and exhibited a distended abdomen that was diffusely tender both spontaneously and on palpation, with guarding and evident clinical signs of peritoneal irritation.

**Figure 1:**
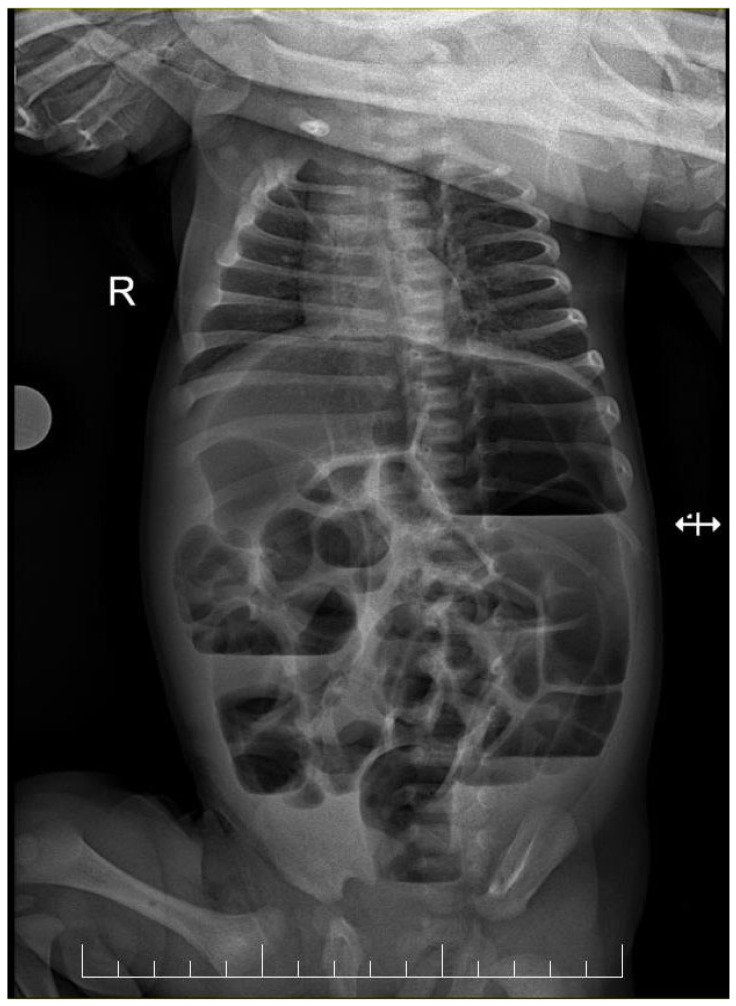
Plain abdominal radiography showing multiple air–fluid levels and significant gaseous bowel distension, suggestive of intestinal obstruction in an 18-day-old neonate. (R: right side; double-headed arrow: horizontal X-ray beam).

Initial laboratory evaluation revealed leukocytosis (15,801/µL) associated with a low C-reactive protein level (0.9 mg/L). Plain abdominal radiography revealed findings suggestive of intestinal obstruction with multiple air–fluid levels, whereas abdominal ultrasonography failed to identify the appendix (Figure 1). Given the clinical presentation suggestive of an acute surgical abdomen, surgical exploration was performed urgently within a few hours of admission without delaying for cross-sectional imaging such as computed tomography (CT) or magnetic resonance imaging (MRI) due to the clinical urgency, concerns regarding radiation exposure in neonates, and the limited availability of MRI in emergency settings in our institution.

**Figure 2:**
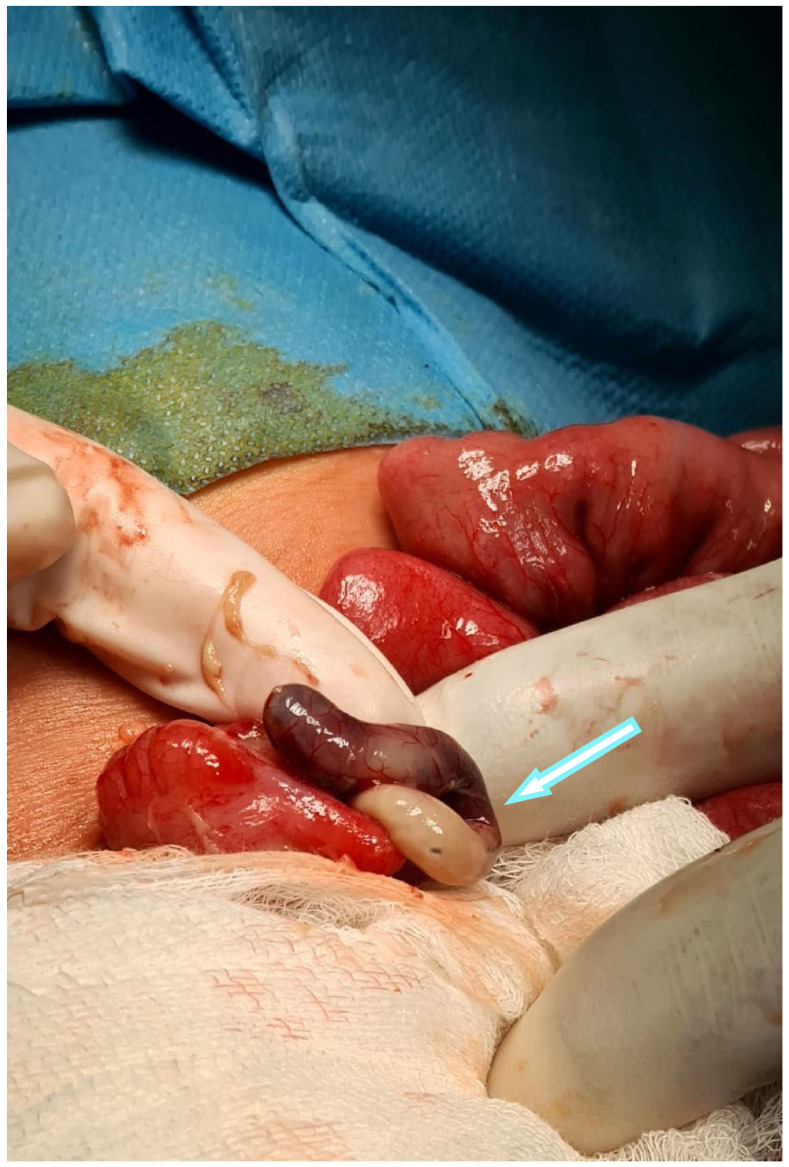
Intraoperative photograph showing a gangrenous perforated vermiform appendix with 240° anticlockwise torsion and black discoloration.

**Figure 3:**
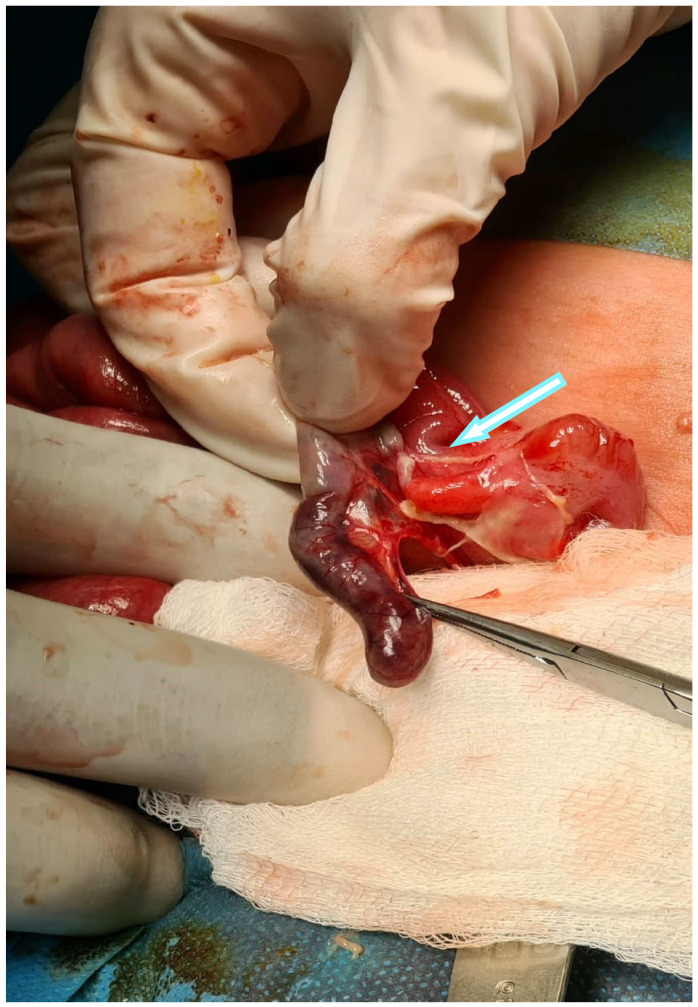
Intraoperative photograph showing a gangrenous vermiform appendix with 240° anticlockwise torsion, black discoloration, and associated fibrinous exudate.

Given the clinical presentation suggestive of an acute surgical abdomen, exploratory laparoscopy was initially planned. Preoperatively, a nasogastric tube was placed for decompression, yielding bilious fluid but failing to significantly reduce the massive bowel distension. Rectal decompression was not performed. A small transverse supraumbilical incision was made, and an open Hasson entry technique was utilized to safely insert a 5 mm primary trocar. Although a limited pneumoperitoneum was initiated, safe and adequate intra-abdominal working space could not be achieved due to the severe bowel distension caused by marked aerocolia. Consequently, laparoscopic visualization was unfeasible, and the procedure was promptly converted to an open approach via a supra- and infraumbilical midline laparotomy to prevent iatrogenic bowel injury. Intraoperative exploration revealed a gangrenous, black-colored vermiform appendix with focal perforation and 240° anticlockwise torsion, associated with fibrinous exudate and multiple mesenteric lymph nodes (Figure 2 and Figure 3). A conventional appendectomy was performed with an operative time of 70 min.

**Figure 4:**
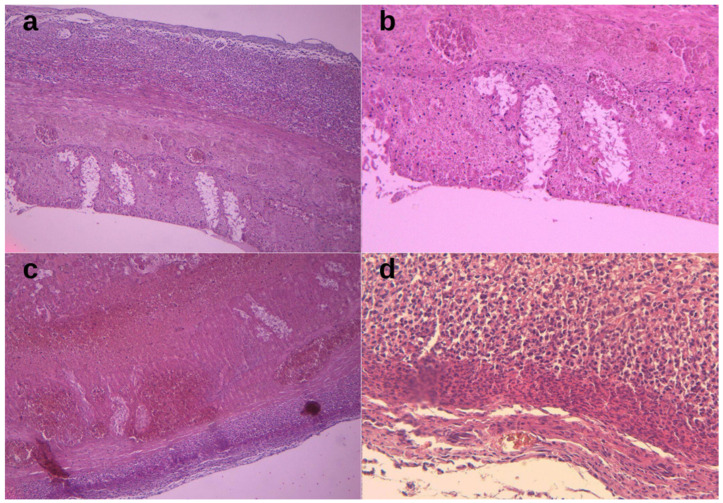
Histopathological examination of the appendicular wall (Hematoxylin-Eosin staining): (**a**) HE 4×: Transmural ischemic necrosis involving all layers, most prominent in the mucosa and submucosa. (**b**) HE 20×: Higher magnification demonstrating complete mucosal and submucosal ischemic necrosis. (**c**) HE 10×: Marked interstitial hemorrhage and severe vascular congestion within the muscularis propria and subserosal layers. (**d**) HE 40×: Intense lymphogranulocytic infiltration within the serosal layer. Panels (**a**,**b**) best illustrate the extensive transmural necrosis, while panel (**c**) highlights the severe vascular congestion and hemorrhage.

The surgical specimen consisted of a 4.5 cm vermiform appendix exhibiting brownish discoloration of the serosa and a lumen obstructed by a coprolith. Microscopic evaluation confirmed ischemic gangrenous changes secondary to appendiceal torsion, characterized by transmural necrosis and significant vascular changes. As shown in Figure 4, the pathological findings included extensive ischemic necrosis of the mucosa and submucosa, significant interstitial hemorrhage within the muscular layers, and a dense lymphogranulocytic infiltrate involving the serosa.

Over the subsequent 24 h postoperatively, repeat laboratory assessment demonstrated the full evolution of the inflammatory syndrome, with the white blood cell count rising to 20,000/µL and a marked rise in C-reactive protein to 140.10 mg/L corroborating the advanced intra-abdominal ischemic and infectious process. The postoperative course was uneventful, and the patient was discharged on postoperative day 5 in good general condition with complete resolution of symptoms.

## 3. Discussion

Torsion of the vermiform appendix represents an exceedingly rare surgical entity, first described by Payne in 1918, with fewer than 100 cases reported in the global literature to date [2,4]. To the best of our knowledge, this is the first documented pediatric case of appendiceal torsion from Romania and, notably, one of the youngest patients reported with this condition, occurring in an 18-day-old neonate. Reporting this case contributes to the regional epidemiological data.

This condition is classified into primary and secondary forms [2,8]. Primary torsion occurs in the absence of any identifiable underlying pathology, whereas secondary torsion develops in association with potential predisposing conditions such as fecaliths, mucoceles, cystic fibrosis, or anatomical anomalies including undescended cecum [2,8,11,12,13].

The pathophysiology of secondary appendiceal torsion in our case may be linked to the presence of the intraluminal coprolith. While a healthy appendix is a lightweight and mobile structure, a dense fecalith may increase the organ’s distal weight, creating a point of inertia. This mass may act as a fulcrum facilitating axial rotation during intestinal peristalsis or sudden changes in the neonate’s body position. Once the rotation exceeds 180°, vascular compromise may occure, beginning with venous and lymphatic obstruction, leading to edema and further distension, and potentially culminating in arterial occlusion and transmural gangrene. However, this sequence remains theoretical, and a definitive causal relationship cannot be established in this single case.

In the present case, the presence of a coprolith within the appendiceal lumen suggests a possible secondary etiology, wherein the fecalith might have potentially acted as a contributing factor facilitating axial rotation [11,12]. The condition is more frequently observed in children than adults, with a reported male predominance [3,8]. The degree of torsion reported in the pediatric literature ranges from 180° to 1080°, most commonly between 180° and 360°, with an anticlockwise direction observed in more than 60% of cases [3,8].

The diagnosis of appendiceal pathology in neonates presents formidable challenges due to the nonspecific nature of clinical manifestations and the rarity of the condition in this age group [14,15]. Neonatal appendicitis itself is exceptionally uncommon, with a historical mortality rate of 23% that has decreased to approximately 3–5% in contemporary series [15,16]. The clinical presentation in our patient—abdominal distension (present in 64.9% of neonates with appendicitis), bilious vomiting, and signs of peritoneal irritation—is characteristic but nonspecific, mimicking more common neonatal surgical emergencies including necrotizing enterocolitis, malrotation with midgut volvulus, and intestinal atresia [15,17,18].

From an anatomical perspective, appendiceal torsion in neonates appears to be uncommon. In newborns, the appendix typically exhibits a funnel-shaped base with a wider communication to the cecum, which generally provides some protection against both luminal obstruction and axial twisting. The occurrence of torsion at just 18 days of life suggests that either a significant predisposing factor—such as the identified coprolith—was present, or there was a localized anatomical variation, such as an abnormally long mesoappendix or a lack of lateral peritoneal fixations (Jackson’s membranes), granting the organ excessive mobility.

The initial laboratory findings in our case demonstrated a dissociation between leukocytosis and C-reactive protein levels, a pattern that has been observed in early neonatal appendicitis and may reflect the immature inflammatory response in this age group [19,20,21]. The subsequent dramatic rise in inflammatory markers over 24 h (C-reactive protein increasing from 0.9 to 140.10 mg/L) underscores the rapid progression of appendiceal pathology in neonates and the narrow therapeutic window available for intervention [19,20,22].

Ultrasonography represents the first-line imaging modality for suspected appendiceal pathology in pediatric patients, though its sensitivity in neonates is limited [14,23]. In a recent case series, the appendix was identifiable on ultrasonography in only 65% of neonates with surgically confirmed appendicitis, with an appendiceal diameter cutoff of ≥3.5 mm providing optimal diagnostic accuracy (AUC 0.89) [14]. The failure to visualize the appendix in our patient is consistent with these reported limitations [14,16]. Plain radiography demonstrating multiple air–fluid levels, as observed in our case, is suggestive of intestinal obstruction but is nonspecific for the underlying etiology [18,24].

Appendectomy remains the standard surgical treatment for appendiceal torsion, which should be performed urgently given the risk of gangrene, perforation, and peritonitis [4,7]. Laparoscopic approaches have been successfully employed in older children and adults; however, in neonates with severe abdominal distension, as in our case, conversion to open surgery may be necessary to ensure safe access and adequate visualization [8,25,26,27]. The operative time of 70 min in our patient reflects the technical complexity inherent to neonatal abdominal surgery, particularly when conversion from laparoscopic to open approach is required. The favorable postoperative course with discharge on postoperative day 5 is consistent with contemporary outcomes in neonatal appendicitis, where mortality rates have significantly improved compared to historical series [15,16,28].

While laparoscopy is increasingly considered the gold standard in pediatric surgery, its application in neonates with massive abdominal distension remains technically demanding. In our patient, the severe aerocolia and resulting high intra-abdominal pressure significantly reduced the working space, making the safe insertion of the primary trocar (Hasson technique) high-risk for iatrogenic bowel injury. The decision to convert to a midline laparotomy was a ‘safety-first’ approach, ensuring adequate visualization and control, which is crucial when dealing with gangrenous tissue and potential perforation in such a small, hemodynamically vulnerable patient. Furthermore, while a transverse abdominal incision may be favored in elective or well-defined scenarios, a midline laparotomy was preferred in this instance due to the preoperative diagnostic uncertainty regarding the exact etiology of the intestinal obstruction. This approach provided optimal surgical exposure and the mandatory flexibility required for a thorough evaluation of the entire abdominal cavity.

The differential diagnosis of acute abdomen with bilious vomiting in neonates encompasses several life-threatening conditions requiring urgent surgical intervention, including malrotation with midgut volvulus, intestinal atresia, necrotizing enterocolitis, incarcerated hernia, and Hirschsprung disease [18,29,30,31]. Appendiceal pathology, whether inflammatory or torsion-related, should be considered in the differential diagnosis of unexplained intra-abdominal sepsis in neonates, particularly when more common etiologies have been excluded [31,32,33,34]. The intraoperative discovery of appendiceal torsion often comes as a surprise to the surgeon, emphasizing the importance of maintaining a broad differential and performing thorough abdominal exploration [4,5,8]. Beyond abdominal symptomatology, appendiceal torsion has also been reported with highly atypical and extra-abdominal clinical presentations, such as acute scrotum in infnts, further highlighting the remarkable heterogeneity and diagnostic challenges associated with this condition [35].

The heterogeneity of appendiceal torsion is best illustrated by the clinical data summarized in Table 1. Our narrative review of the 34 previously reported pediatric cases reveals a predominantly male distribution and a wide range of axial rotation (180–1080°), with the anticlockwise direction being the most frequent. By integrating our findings with the available literature, and to the best of our knowledge, this report not only identifies the first Romanian neonatal case but also appears to represent the youngest reported neonatal case of appendiceal torsion, occurring in an 18-day-old patient. As evidenced by the table, the persistent diagnostic challenge lies in the lack of pathognomonic clinical or radiological signs, with surgery remaining the only definitive diagnostic and therapeutic tool. Taken together, Table 1 includes the 34 previously reported pediatric cases as well as the present case, resulting in a total of 35 pediatric cases of appendiceal torsion.

The histopathological examination in our case revealed a gangrenous appendix with brownish discolored serosa and luminal coprolith, findings consistent with vascular compromise, in the context of torsion and luminal obstruction; however, a definitive causal sequence cannot be established in this single case. The presence of a coprolith supports the hypothesis of secondary torsion, wherein appendiceal distension and increased weight may have predisposed to axial rotation [8,11]. Similar findings have been reported in other pediatric cases of appendiceal torsion, where fecaliths were identified as potential contributing factors [8,61].

The extreme rarity of appendiceal torsion precludes the development of evidence-based diagnostic algorithms or management guidelines [4,8]. Case reports and small series remain the primary source of clinical knowledge regarding this entity [2,4,8]. Future multicenter collaborations may help elucidate predisposing factors, optimal diagnostic approaches, and outcomes in this condition [15,32].

## 4. Conclusions

Torsion of the vermiform appendix is an exceptionally rare condition in the pediatric population and even more uncommon in neonates, with very few cases reported in the literature [4,8]. To the best of our knowledge, the present case represents the first documented pediatric case of appendiceal torsion from Romania and is among the youngest patients reported globally. Because its clinical presentation is highly nonspecific and frequently mimics other causes of neonatal acute abdomen, preoperative diagnosis is extremely challenging and, in most cases, impossible [4,8,31,32,33,49]. Imaging studies are often inconclusive, and a definitive diagnosis is typically established intraoperatively [14,16]. Early surgical intervention remains essential, both for diagnosis and treatment, with appendectomy providing definitive treatment [4,14]. Prompt management is crucial to prevent complications such as gangrene and peritonitis, particularly in this vulnerable age group [14,62].

Although rare, appendiceal torsion should be considered in the differential diagnosis of neonatal acute abdomen, and timely surgical exploration is key to achieving a favorable outcome [4,16,19].

## Figures and Tables

**Table 1:** Summary of reported cases of appendiceal torsion in pediatric patients aged <18 years (1959–2026): Demographics, torsion dynamics, preoperative diagnosis, and operative approach.

No.	Authors/Year	Age/ Gender	Degree/ Direction of Torsion	Symptoms Nausea/ Vomiting/ Fever °C	Length (cm)	Etiology of Torsion	Preoperative Diagnosis	Type of Surgery
1	Carter/1959 [36]	8 years/F	>360/AC	Vomiting/38.3	UD	UD	UD	OS
2	Carter/1959 [36]	16 years/M	>360/AC	Nausea/37.4	UD	UD	UD	OS
3	Ghent WR. et al., 1966 [37]	12 years/M	360/C	Nausea/37.6	7	Primary torsion	UD	OS
4	Finch DR., 1974 [38]	12 years/M	270/AC	Vomiting/37.2	UD	UD	UD	OS
5	Willan PT et al., 1983 [39]	4 years/M	720/AC	Vomiting/37.3	7	UD	Acute appendicitis	OS
6	Dewan PA. et al., 1986 [40]	3 years/M	720/AC	Vomiting/37.9	7	Primary torsion	UD	OS
7	Dewan PA. et al., 1986 [40]	6 years/F	1080/C	Vomiting/37.4	7	Primary torsion	UD	OS
8	Dewan PA. et al., 1986 [40]	16 years/M	UD	No fever	UD	UD	UD	OS
9	Waters DJ. et al., 1986 [41]	3 years/M	720/UD	Vomiting/38.9	UD	UD	UD	OS
10	Lowry WL. et al., 1986 [42]	2 months/M	UD	UD	UD	UD	UD	OS
11	Yeung CK. et al., 1991 [9]	50 days/M	UD	High fever	UD	UD	Generalized peritonitis	OS
12	Merrett ND. et al., 1992 [35]	14 years/M	720/AC	Vomiting/37.5	14	Primary torsion	Acute appendicitis	OS
13	Glichrist, 1995 [43]	9 years/M	360/AC	Vomiting/37.2	9	Primary torsion	UD	OS
14	Val-Bernal et al., 1996 [3]	6 years/M	>360/AC	Vomiting/37.5	13.5	Primary torsion	Acute appendicitis	OS
15	Uroz-Tristan et al., 1998 [44]	5 years/M	360/AC	Vomiting	15	Primary torsion	Torsion or mucocele	OS
16	Oguzkurt et al., 2004 [45]	2 years/M	270/AC	Vomiting/38	10	Secondary torsion/duplicated colon and appendix	UD	OS
17	Gopal K et al., 2005 [46]	9 years/M	720/UD	Vomiting	5	PT	Acute appendicitis	LS
18	Sarin YK. et al., 2006 [47]	9 years/M	270/C	Vomiting/37.7	8	PT	UD	OS
19	Baeza-Herrera C. et al., 2006 [48]	2 months/F	360/AC	37.5	UD	ST/ileocecal intussusception	Ileocecal intussusception	OS
20	Montes-Tapia, 2009 [49]	3 years/M	1080/AC	Vomiting/37.5	UD	PT/undescended and mobile cecum	Acute abdomen	LS
21	Perger L. et al., 2011 [50]	11 weeks/F	360/AC	37.5	UD	PT	Acute appendicitis	LS
22	D’Souza GF. et al., 2011 [51]	2 years/M	UD	Vomiting/38.3	6.5	PT	Acute appendicitis	LS
23	Adi MY. et al., 2014 [52]	4 years/M	720/AC	Vomiting/fever	7	PT	Acute appendicitis	LS
24	Ioannis V. et al., 2017 [53]	4 years/M	270/C	Vomiting/fever	UD	PT	Acute appendicitis	LS
25	Hirpara DH. et al., 2018 [54]	2 years/M	720/C	Vomiting/37.5	7.5	ST/lymphoid hyperplasia	Acute appendicitis or Meckel ‘s diverticulitis	OS
26	Endo et al., 2020 [4]	4 years/M	720/C	Vomiting/37.2	8	PT	Acute appendicitis	LS
27	Samuk I. et al., 2021 [55]	40 months/M	UD	Vomiting/UD	UD	UD	Acute appendicitis	OS
28	Samuk I. et al., 2021 [55]	23 months/M	1080/UD	Vomiting/UD	UD	UD	Perforated appendicitis with appendicular abscess	OS
29	Chiarenza SF et al., 2021 [8]	5 years/M	720/AC	Vomiting/38	6–7	PT/narrow appendicular mesentery and movable cecum	Acute appendicitis	LS
30	Hashimi, H. et al., 2021 [56]	16 years/M	720/AC	UD	UD	ST/Mucocele	Localized peritonitis	LS
31	Medinskiy PV et al., 2023 [57]	10 years/M	UD	Vomiting/no fever	7	PT	Acute appendicitis	LS
32	Mili T et al., 2023 [58]	11 years/M	360/C	Vomiting/37.5	8	PT	Acute appendicitis	OS
33	Alfarsi MA et al., 2024 [59]	9 years/F	360/AC	Vomiting/fever	5	ST/Foreign body	Acute appendicitis	LS
34	Zheng C et al., 2024 [60]	11 weeks/F	1080/AC	No fever	3	PT	Acute abdomen	OS
35	Presented case 2026	18 days/F	240/AC	Vomiting/ 36.9	4.5	ST/Coprolith	Intestinal obstruction	Converted from LS to OS

Note: AC = anticlockwise; C = clockwise; UD = undetermined; PT = primary torsion; ST = secondary torsion; OS = open surgery; LS = laparoscopy.

## Data Availability

The original contributions presented in this study are included in the article. Further inquiries can be directed to the corresponding author.

## Abbreviations

The following abbreviations are used in this manuscript:

AC	Anticlockwise
APC	Article Processing Charge
AUC	Area Under the Curve
C	Clockwise
CRP	C-reactive Protein
LS	Laparoscopy
NEC	Necrotizing Enterocolitis
NR	Not Reported
OS	Open Surgery (Laparotomy)
PT	Primary Torsion
ST	Secondary Torsion
UD	Undetermined
US	Ultrasonography
WBC	White Blood Cell Count
